# Eccentric Cycling Training Improves Erythrocyte Antioxidant and Oxygen Releasing Capacity Associated with Enhanced Anaerobic Glycolysis and Intracellular Acidosis

**DOI:** 10.3390/antiox10020285

**Published:** 2021-02-13

**Authors:** Yu-Chieh Huang, Mei-Ling Cheng, Hsiang-Yu Tang, Chi-Yao Huang, Kuan-Ming Chen, Jong-Shyan Wang

**Affiliations:** 1 Department of Physical Therapy, College of Medical and Health Science, Asia University, Taichung 413, Taiwan; yuchieh@asia.edu.tw; 2 Metabolomics Core Laboratory, Healthy Aging Research Center, Chang Gung University, Taoyuan 333, Taiwan; chengm@mail.cgu.edu.tw (M.-L.C.); tangshyu@gmail.com (H.-Y.T.); 3 Clinical Metabolomics Core Laboratory, Chang Gung Memorial Hospital, Taoyuan 333, Taiwan; 4 Department of Biomedical Sciences, College of Medicine, Chang Gung University, Taoyuan 333, Taiwan; 5 Healthy Aging Research Center, Graduate Institute of Rehabilitation Science, Medical Collage, Chang Gung University, Taoyuan 333, Taiwan; kjes9210@hotmail.com (C.-Y.H.); ttlike76@gmail.com (K.-M.C.); 6 Heart Failure Center, Department of Physical Medicine and Rehabilitation, Keelung Chang Gung Memorial Hospital, Keelung 204, Taiwan; 7 Research Center for Chinese Herbal Medicine, College of Human Ecology, Chang Gung University of Science and Technology, Taoyuan 333, Taiwan

**Keywords:** eccentric exercise, redox status, erythrocyte, metabolism

## Abstract

The antioxidant capacity of erythrocytes protects individuals against the harmful effects of oxidative stress. Despite improved hemodynamic efficiency, the effect of eccentric cycling training (ECT) on erythrocyte antioxidative capacity remains unclear. This study investigates how ECT affects erythrocyte antioxidative capacity and metabolism in sedentary males. Thirty-six sedentary healthy males were randomly assigned to either concentric cycling training (CCT, *n* = 12) or ECT (*n* = 12) at 60% of the maximal workload for 30 min/day, 5 days/week for 6 weeks or to a control group (*n* = 12) that did not receive an exercise intervention. A graded exercise test (GXT) was performed before and after the intervention. Erythrocyte metabolic characteristics and O_2_ release capacity were determined by UPLC-MS and high-resolution respirometry, respectively. An acute GXT depleted Glutathione (GSH), accumulated Glutathione disulfide (GSSG), and elevated the GSSG/GSH ratio, whereas both CCT and ECT attenuated the extent of the elevated GSSG/GSH ratio caused by a GXT. Moreover, the two exercise regimens upregulated glycolysis and increased glucose consumption and lactate production, leading to intracellular acidosis and facilitation of O_2_ release from erythrocytes. Both CCT and ECT enhance antioxidative capacity against severe exercise-evoked circulatory oxidative stress. Moreover, the two exercise regimens activate erythrocyte glycolysis, resulting in lowered intracellular pH and enhanced O_2_ released from erythrocytes.

## 1. Introduction

Endurance training is essential to maximally improve cardiopulmonary fitness and delay the disease process. However, this may be intolerable due to the overload of the cardiopulmonary system to elderly individuals or patients with chronic diseases, traditional concentric work at usual training intensity [1]. Eccentric endurance training has the ability to overcome these limitations because of less respiratory requirement and metabolic oxygen, as well as lower heart rate (H), cardiac index and blood lactate concentration than concentric type at equivalent workload [2]. The benefits of using eccentric cycling training (ECT) in chronic heart failure patients [3], elderly individuals [4] and chronic obstructive pulmonary disease [5] have been confirmed. Conventionally, most studies have focused on the contribution to elicit neuromuscular adaptations of eccentric work [6]; nevertheless, a recent study further demonstrated that either acute bout of concentric or eccentric cycling at moderate intensity induced increased enzymatic antioxidant capacity and decreased oxidative stress markers [7]. Moreover, ECT induces greater fat utilization compared to concentric cycling training (CCT) at a fixed workload [8] and greater fat loss in obese adolescents [9]. Therefore, the different cardiopulmonary loading and metabolic oxygen demands in ECT and CCT may result in distinct changes in antioxidative metabolism and O_2_ release adaptations [10]. However, there is very limited evidence regarding these mechanisms of chronic physiological responses to eccentric cycling [11].

Erythrocytes are vital to humans because of their abundance and the irreplaceable function they have of delivering O_2_. However, they are susceptible to sustained free radical damage during circulation, which impairs their O_2_ release capacity and reduces their lifespan [12]. Previous studies have reported that blood antioxidation capacity is impaired with acute exercise [13]; in contrast, regular exercise may increase antioxidative capacity [14]. The lower oxygen and energy consumed in ECT may avoid repeated, excessive exposure to oxidative stress, which progressively impair the erythrocyte [15]. However, whether this lower metabolic stress in comparable ECT might be enough to elicit physiological adaptations as CCT or not is another concern [16]. To date, the adaptations of the antioxidation capacity and regulatory mechanism of erythrocytes under different exercise regimens remain unclear. Here, we identified the key regulatory mechanisms using metabolomics profiling technology.

When exercising, erythrocytes must accelerate O_2_ release into peripheral tissue according to the Bohr effect [17] and enhance the demand for glycolytically derived ATP to restore intracellular ion balances. This process is at a constant rate when ATP consumption is steady, but the activity of the process changes rapidly in response to enhanced ATP utilization [18]. Importantly, erythrocytes are also exposed to dramatically enhanced oxidative stress that must be controlled by accelerated production of reducing equivalents derived from the pentose phosphate pathway (PPP), which is the sole source of NADPH and produces GSH as an antioxidant. In the sickle cells, the impaired antioxidant capacity leaves to a loss of glycolysis and the PPP shifting mechanism control and further homeostasis rupture, contributing to a decreased lifespan of cells [19]. Moreover, altering glycolytic pathway dominance has been demonstrated to limit antioxidation capacity under hypoxia [20]. Therefore, exercise may introduce continuous substrate competition between the main glycolysis pathway and the PPP, although this needs to be further elucidated.

2,3-BPG is a strong allosteric modulator that leads to O_2_ unloading [21]. However, the generation of 2,3-BPG bypasses the main phosphoglycerate kinase reaction so that the overall production of ATP per mole of glucose is decreased to zero. GSH de novo synthesis is ATP dependent and is therefore impaired when the stocks of intracellular ATP are depleted. In addition, lactate is the only end product of glycolysis in erythrocytes, and it also helps create a low pH value environment to decrease Hb-O_2_ affinity [22] and influence GSH synthesis [23]. Therefore, one of the biggest puzzles regarding erythrocyte metabolism during exercise is how the programming of erythrocyte glucose metabolism, 2,3-BPG production, and antioxidative capacity is regulated.

To address the abovementioned questions, this study elucidated the pathways underlying the regulation of the main glycolysis and the PPP and explored the effects of oxidation and antioxidation capacity in erythrocytes after six weeks of interventions. In addition, we also investigated the capacity for O_2_ release under different lactate concentrations under hypoxic and normoxic conditions. The aim of this study was to provide direct evidence that both ECT and CCT induce metabolic adaptations within erythrocytes that counteract the high oxidative stress evoked by vigorous exercise.

## 2. Materials and Methods

### 2.1. Subjects

The intervention was performed in accordance with the Declaration of Helsinki and was approved by the Institutional Review Board of Chang Gung Memorial Hospital in Taiwan. A total of 36 sedentary males who were nonsmokers, nonusers of medication/vitamins, and free of any cardiopulmonary/hematological risks were recruited from Chang Gung University, Taiwan. No subject had performed regular exercise (i.e., exercise frequency once per week, duration < 20 min) for at least 1 year before the experiment. All subjects provided informed consent after the experimental procedures were explained. These subjects were randomly divided into three groups: the concentric cycling training (CCT, *n* = 12), the eccentric cycling training (ECT, *n* = 12), and the control (CTL, *n* = 12) groups. All subjects arrived at the testing center at 9:00 AM to eliminate any possible circadian effect. Participants were instructed to fast for at least 8 h and to refrain from strenuous physical exercise for at least 48 h before sampling.

### 2.2. Protocol and Interventions

Both the CCT and ECT groups performed exercise regimens on a stationary bicycle ergometer (CCT: Corival 400, Lode; ECT: custom-built cycle ergometer) 5 times a week for 6 weeks. For comparison, the CTL group did not perform any exercise, but their physical activities and daily diet were carefully documented.

All subjects reported their daily activities and nutrition intakes via questionnaires throughout the experiment. The participants were instructed to refrain from extra regular exercise until the end of this study. Moreover, the compliance rates for all three interventions were 100%.

**Figure 1:**
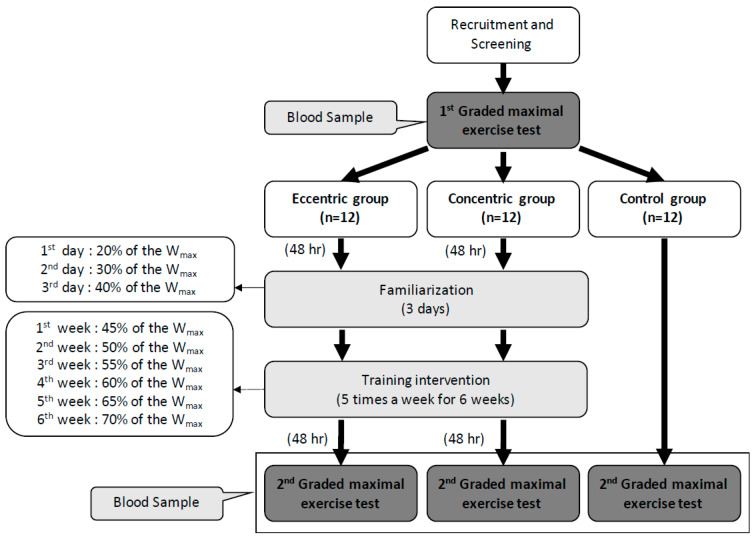
Design of the experiment and the training intensity of eccentric and concentric groups in each week. W_max_: the maximal workload of the first graded maximal exercise test.

The graded exercise test (GXT) was performed 48 h before and after the intervention. Both the CCT and ECT groups had a 3 day familiarization program upon initiation of training. The exercise intensity was set at 20%, 30%, and 40% of the maximal workload (W_max_) on each day. The first week’s intensity was set at 45% W_max_ and progressively increased 5% W_max_ per week until 70% W_max_ was obtained in the sixth week. Each training session contained a 6-min warm-up phase (3 min at 0% and 3 min at 30% W_max_), 30-min training period and 6-min cool-down phase (3 min at 30% and 3 min at 0% W_max_) (Figure 1). The training groups were asked to record their daily activities and nutritional intake using the short form of the international physical activity questionnaire and a written diet record, respectively. Subjects were asked to refrain from regular extra exercise until the end of the study. The participant compliance rate was 100% throughout this study.

### 2.3. Graded Exercise Tests

To assess aerobic capacity, a GXT was performed on a cycle ergometer (Corival 400, Lode B.V., Groningen, The Netherlands). After a 5-min baseline resting period, a 2-min warm-up period (60 rpm, unloaded pedaling) was initiated, followed by incremental work (30 Watt increase for each 3 min) until exhaustion (i.e., progressive exercise to VO_2max_). Minute ventilation (V_E_), oxygen consumption (VO_2_), and carbonic dioxide production (VCO_2_) were measured for each breath by using a computer-based system (MasterScreen CPX, CareFusion, Franklin Lakes, NJ, USA). The criteria used to define VO_2max_ were as follows: (i) the level of VO_2_ increased by < 2 mL/kg/min over at least 2 min; (ii) H exceeded its predicted maximum; (iii) the respiratory exchange ratio (RER) exceeded 1.2; and (iv) the venous lactate concentration was >8 mM, which was consistent with the guidelines of the American College of Sports Medicine for exercise testing [24]. Additionally, the ventilation threshold (VT) was determined when VE/VO_2_ increased without a corresponding increase in the V_E_-to-VCO_2_ ratio and when end-tidal PO_2_ increased without a decrease in end-tidal PCO_2_ or a deviation from linearity for V_E_.

### 2.4. Erythrocyte Isolation and Blood Collection

At rest and immediately after the GXT, a 10 mL blood sample was collected from the antecubital vein via clean venipuncture (20-gauge needle) and added to a tube with ethylenediaminetetraacetic acid (EDTA, 4 mM). Blood cells were counted by using a Sysmex SF-3000 cell counter (GMI Inc., Ramsey, MN, USA), and the blood pH and lactate concentration were tested by an i-STAT handheld blood analyzer (Abbott Point of Care Inc., Princeton, NJ, USA). Erythrocytes were isolated from whole blood by centrifugation (1000× *g* for 15 min at RT), the supernatant was discarded, and the buffy coat was discarded, followed by three washing steps in PBS with 0.1% (*w/v*) glucose (Sigma). The erythrocyte count was adjusted to 1 × 10^4^ cells/µL using PBS.

### 2.5. Measurement of Reactive Oxygen Species (ROS) Production

2′, 7′-Dichlorofluorescin diacetate (DCFDA) is a fluorogenic dye that measures hydroxyl, peroxyl and other ROS activities within the cell. This study used a DCFDA Cellular ROS Detection Assay Kit (ab113851, Abcam) to measure intracellular ROS according to the manufacturer’s protocol. DCFDA-labeled erythrocytes were treated with different concentrations of tert-butyl hydroperoxide (TBHP) (5 mM, 10 mM, 50 mM and 100 mM) at 37 °C for 30 min. TBHP mimics ROS activity to oxidize DCFDA to fluorescent DCF. Finally, the mean fluorescence intensities obtained from 50,000 erythrocytes were measured using FACSCalibur (Becton Dickinson, NJ, USA). All samples were analyzed in triplicate, and the intraassay CV was 4.1 ± 0.7%.

### 2.6. Erythrocyte Intracellular pH

As our previous study presented [25], erythrocytes were loaded with the fluorescent pH indicator carboxy SNARF-1 (1 µM, Invitrogen) at 37 °C for 30 min in the dark and then washed with HBSS (Sigma) at 2500× *g* for 5 min. SNARF-1-loaded cells were incubated with pH-controlled normal K^+^-balanced buffer (137.9 mM NaCl, 5.33 mM KCl, 0.441 mM KH2PO4, 4.17 mM NaHCO3, 0.338 mM Na2HPO4, 5.56 mM glucose, and 20 mM HEPES, pH = 7.5) and high K^+^-balanced buffer at different pH values (43.7 mM NaCl, 100 mM KCl, 0.441 mM KH2PO4, 4.17 mM NaHCO3, 0.338 mM Na2HPO4, 5.56 mM glucose, and 20 mM HEPES, pH = 6.8, 7.0, 7.2, 7.4, 7.6, 7.8, and 8.0) containing 10 µM nigericin (Invitrogen). The pH was always adjusted at RT prior to use. The pH-dependent spectral shifts exhibited by SNARF-1 allowed calibration of the pH response in terms of the ratio of fluorescence intensities measured at two different wavelengths, FL2 and FL3, in a FACSCalibur (λ1 = 580 nm and λ2 = 600~640 nm and fixed excitation at 514 nm), as described in the manufacturer’s protocol (Invitrogen). All samples were analyzed in triplicate, and the intraassay CV was 3.7 ± 0.9%. The equation is as follows:

(1) $${\mathrm{pH}=\mathrm{pK}}_{a}{-\log}_{10}\left[\frac{{R-R}_{B}}{R_{A}-R}\times \frac{F_{B(\lambda 2)}}{F_{A(\lambda 2)}}\right]$$

pKa values: 7.5 for carboxy SNARF-1;

R: The ratio Fλ1/Fλ2 of fluorescence intensities (F) measured at λ1 and λ2;

The subscript A represents the limiting values at the acidic endpoints of the titration;

The subscript B represents the limiting values at the basic endpoints of the titration.

### 2.7. Oxygen Release Efficacy in Erythrocytes

High-resolution respirometry, an Oroboros Oxygraph-2 K (Oroboros Instruments, Innsbruck, Austria), was used to measure the O_2_ pressure (mmHg) and O_2_ flux per volume (pmol·s^−1^·mL^−1^) of erythrocytes at 0-, 1-, and 4 mM lactate during hypoxia (PO_2_ = 20 ± 3 mmHg) and normoxia (PO_2_ = 147 ± 3 mmHg) in HBSS medium, respectively. Hypoxic conditions were prepared by gassing with nitrogen (N_2_) gas. After heating at 37 °C and equilibration and calibration for the target PO_2_ conditions, 2 × 10^6^ isolated erythrocytes were added, and after the signaling stabilized, 2 and 6 µL of 1 M lactic acid (Sigma) were sequentially added to form 1 and 4 mM lactic acid environments to simulate the rest and lactate threshold conditions, respectively (Figure S1). All samples were analyzed in triplicate, and the intraassay CV was 3.9 ± 0.8%.

### 2.8. Sample Preparation for Targeted Metabolite Identification and Quantification

Fifteen randomly selected samples (ECT = 5, CCT = 5, and CTL = 5) were quantified for the target metabolite. Erythrocytes (6 × 10^8^) were lysed in 200 µL of ddH_2_O containing 100 ppb of debrisoquine sulfate (Sigma) as an internal control. The lysate was extracted in 800 µL of warmed methanol. The sample was incubated at RT for 15 min to precipitate proteins and centrifuged at 16,000× *g* for 30 min at 4 °C. The supernatant was transferred to a new tube, dried under nitrogen gas, and stored at −80 °C. Prior to analysis, the sample was dissolved in 200 µL of ddH_2_O containing 0.1% formic acid. The procedure was carried out according to the method of Tang et al. [26].

### 2.9. Target Metabolite Analysis of Glycolysis Intermediates

All samples were analyzed using ultrahigh-performance liquid chromatography (UHPLC) coupled with Xevo TQ-S MS (Waters Corp., MA USA) as previously described with modifications [27]. MS was performed in negative-ion multiple-reaction-monitoring (MRM) mode. For tuning purposes, a single analysis standard dissolved in a mixture of water/methanol 50:50 (*v/v*) was infused at a flow rate of 10 μL/min. The desolvation gas flow was set at 1000 L/h at a temperature of 500 °C, and the source temperature was set at 150 °C. The capillary voltage and cone voltage were set to 1.3 kV and 25 V, respectively. For chromatographic separation, a BEH C18 (100 mm × 2.1 mm, 1.7 µm; Waters Corp, MA, USA) was used with eluent A (10 mM tributylamine aqueous solution with 15 mM acetic acid) and eluent B (50% acetonitrile containing 10 mM tributylamine and 15 mM acetic acid), the flow rate was 0.3 mL/min, and the column temperature was set at 25 °C. The gradient profile was as follows: linear-gradient 99–98% solvent B, 8 min; 12% solvent B, 2 min; 55% solvent B, 2 min; and 99% solvent B, 2 min. The column was then re-equilibrated for 4 min. QC samples were analyzed during the analytical runs. All samples were analyzed in triplicate, and the intraassay CV was 3.4 ± 0.6%.

### 2.10. Senescence-Related Biological Markers and Methemoglobin Concentrations in Erythrocytes

The erythrocyte suspension (1 × 10^4^ cells/µL) was incubated with saturating concentrations of monoclonal anti-human CD47 antibody (BioLegend) or monoclonal anti-human CD147 antibody (eBioscience) conjugated with fluorescein isothiocyanate (FITC) in the dark for 30 min at 37 °C. The mean fluorescence intensity (MFI) obtained from 50,000 erythrocytes was measured by a FACSCalibur (Becton Dickinson, NJ USA). Human methemoglobin (met-Hb) ELISA kit (CSB-E09493 h, CUSABIO, Huston, TX USA) obtained from CUSABIO was used according to the manufacturers’ instructions. All samples were analyzed in triplicate, and the intraassay CV was 4.4 ± 0.7%.

### 2.11. Statistical Analysis

Data were analyzed using the statistical software SPSS 22.0 (SPSS, Chicago, IL USA), and continuous data are expressed as the means ± SEM. Nonparametric results were examined by the Mann–Whitney U test and Wilcoxon signed ranked test. Parametric results were tested by two-way repeated-measures ANOVA (group × time points) and the Newman–Keuls post hoc test to identify significant changes pre- vs. postintervention and pre- vs. post-graded exercise tests. Correlations were measured by Pearson’s correlation coefficient. The level of statistical significance was *p* < 0.05.

## 3. Results

### 3.1. Cardiopulmonary Fitness and Hematological and Blood Gas Parameters

There were no differences in anthropometric characteristics, hematological parameters, blood pH, lactate concentration or exercise performance among the groups at baseline (Table 1). Following 6 weeks of training, both the CCT and ECT groups demonstrated increases in work rate, VE, and VO_2_ at the ventilation threshold (VT). Moreover, CCT was superior to ECT for enhancing the work rate and VO_2_ at VT. At the peak performance, only CCT enhanced the VEmax and VO_2_ max, while ECT only resulted in an improvement in the work rate (Table 1). However, 6 weeks of the CTL did not influence hematological parameters or cardiopulmonary responses to a GXT (Table 1).

### 3.2. Pain Scale Scores, Heart Rate and Systolic Blood Pressure during the Training Period

**Figure 2:**
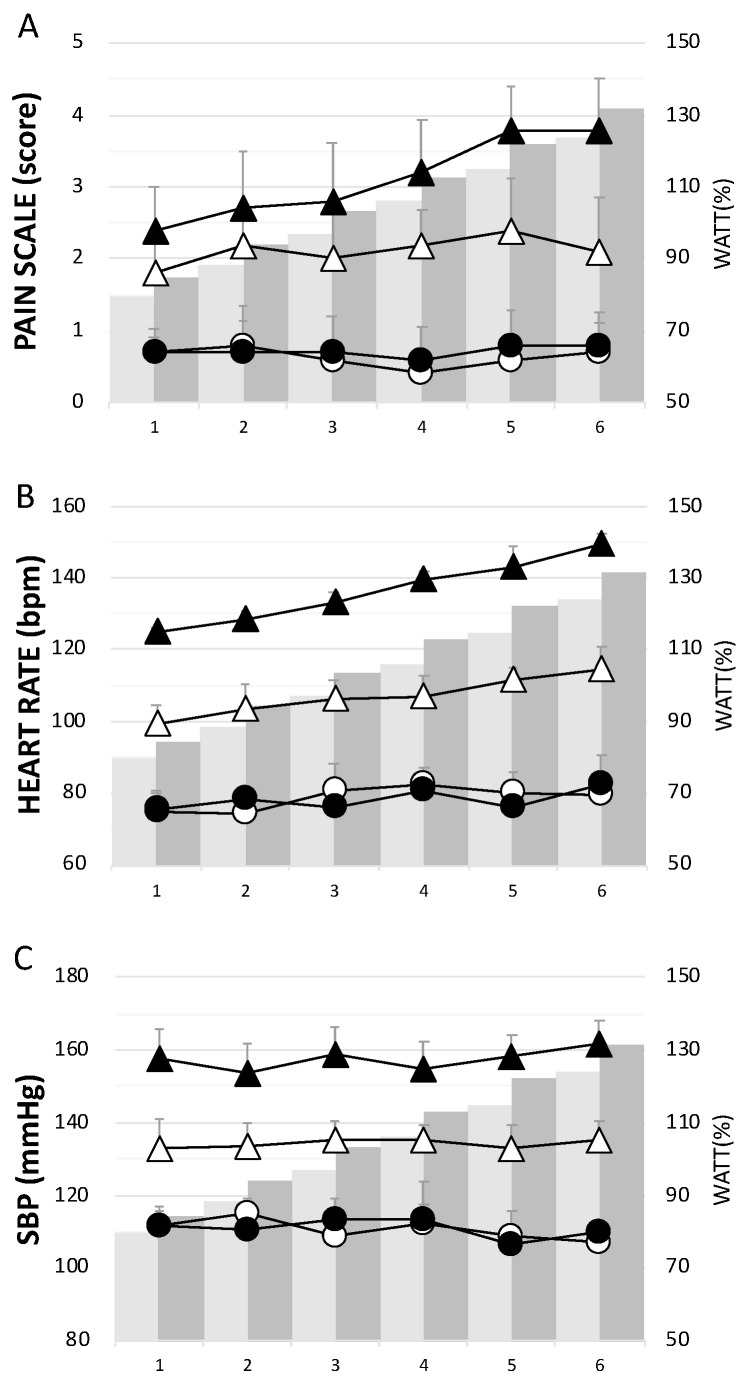
The physiological responses in each week during the training period. (**A**) pain scale score, (**B**) heart rate, and (**C**) systolic blood pressure. Whites— eccentric cycling training (ECT); Blacks— concentric cycling training (CCT); Dots—before training; Triangles—after training. Values were mean ± SEM.

The CCT group had significantly higher levels of pain, H, and SBP than the ECT group throughout the 6 week training period (Figure 2). Concerning the assessment of pain or soreness, the specific pain scale score was close to zero in both groups before each training session (Figure 2A).

### 3.3. Erythrocyte Senescence-Related Markers and Antioxidation Capacity

**Figure 3:**
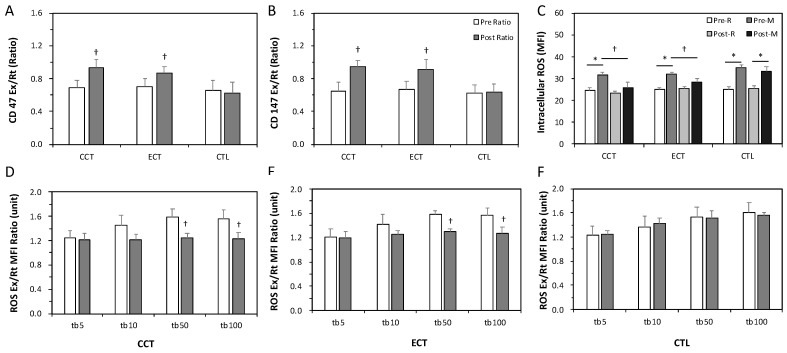
Effects of various ECT and CCT on the erythrocyte senescence-related biomarkers, intracellular reactive oxygen species (ROS) level and the ROS dose-response. (**A**) the ratio of post-graded exercise test (GXT) to pre-GXT in CD47, (**B**) the ratio of post-GXT to pre-GXT in CD147, (**C**) the intracellular ROS level among three groups; the ratio of Ex to Rt ROS response in erythrocytes treated with different concentrations of tert-butyl hydroperoxide (tb): (**D**) the CCT group, (**E**) the ECT group, and (**F**) the control (CTL) group. **Pre**, pre-intervention; Post, post-intervention; M or Ex, immediately after a GXT; R or Rt, at rest. * *p* < 0.05, R vs. M; † *p* < 0.05, Pre vs. Post. Values were mean ± SEM.

The ratios of Ex to Rt in CD147 and CD47 cells were less than 1 before training, indicating enhanced senescence in erythrocytes due to an acute GXT. After the interventions, these ratios significantly increased to nearly 1 in response to a GXT (Figure 3A,B). Intracellular ROS levels were significantly increased after an acute GXT among the three groups; however, both training groups had lower ROS production related to the GXT after training (Figure 3C). Furthermore, as Figure 3D,F shows, a higher TBHP concentration induced greater ROS generation. Nevertheless, the two exercise regimens significantly diminished the extent of ROS generation under 50 and 100 mM TBHP conditions (Figure 3D,E). No alteration was observed in the CTL group (Figure 3F).

### 3.4. Target Metabolite Analysis of Glycolysis and Pentose Phosphate Pathway Intermediates

**Figure 4:**
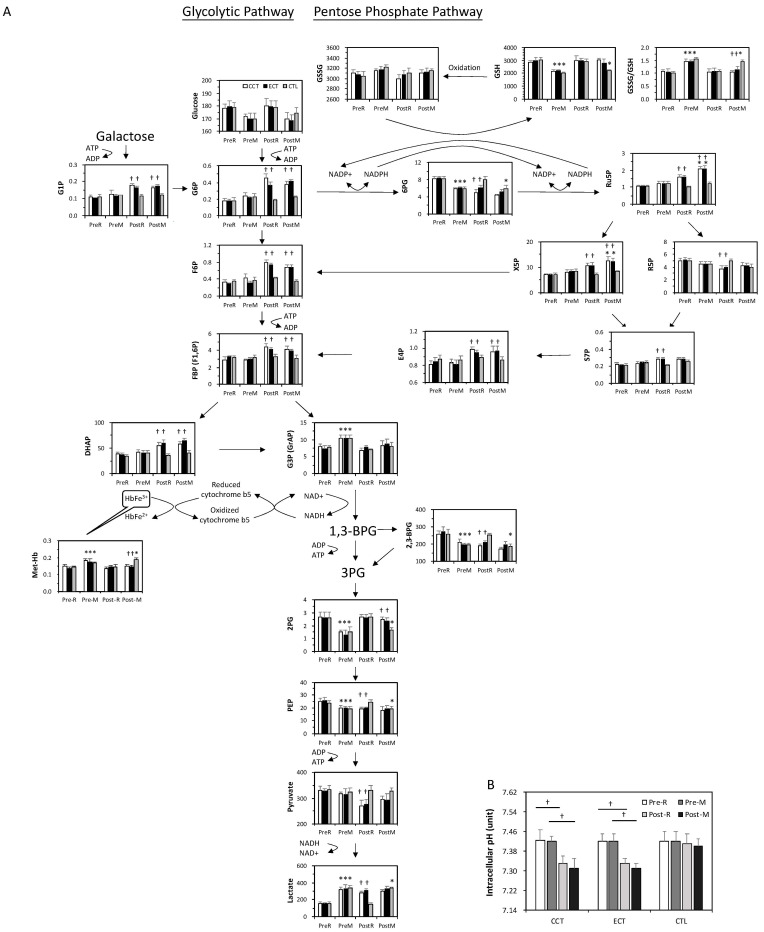
The target metabolite analysis of glycolysis and the pentose phosphate pathway intermediates in erythrocytes in ECT and CCT. (**A**) Levels of metabolites in pentose phosphate pathway and glycolytic pathway (*n* = 5) (**B**) the intracellular pH of erythrocytes. **Pre**, pre-intervention; **Post**, post-intervention; R, at rest; M, immediately after a GXT; * *p* < 0.05, R vs. M; † *p* < 0.05, Pre vs. Post. Values were mean ± SEM.

At baseline, the acute GXT showed greater glucose consumption and was accompanied by a series of unchanged levels of downstream metabolites until G3P (Figure 4A). The GSSG/GSH ratio significantly increased following an acute GXT, indicating an accumulation of oxidative stress. In addition, 2,3-BPG was depleted after the GXT.

Both glycolysis and the PPP were upregulated due to exercise training. Although no change was observed in the glucose level, the downstream metabolites were markedly elevated even under resting conditions. Interestingly, although 2,3-BPG was lower after training, the end product of glycolysis, lactate, was dramatically increased. The PPP flux was also facilitated, as evidenced by a decrease in 6PG and a constant GSSG/GSH ratio. Furthermore, the higher levels of Ru5P and GSH suggested a significant enhancement of GSH biosynthesis under stress. X5P and E4P are intermediates between the PPP and glycolysis, which was dramatically enhanced in both training groups.

In addition, the level of met-Hb was significantly increased by the acute GXT compared to the rest among the three groups, whereas the extent of elevation was diminished after training. The intracellular pH in erythrocytes did not change relative to the GXT before training, whereas both CCT and ECT lowered the pH value after 6 weeks (Figure 4B).

### 3.5. Erythrocyte O_2_ Release Capacity in Normal Conditions

**Figure 5:**
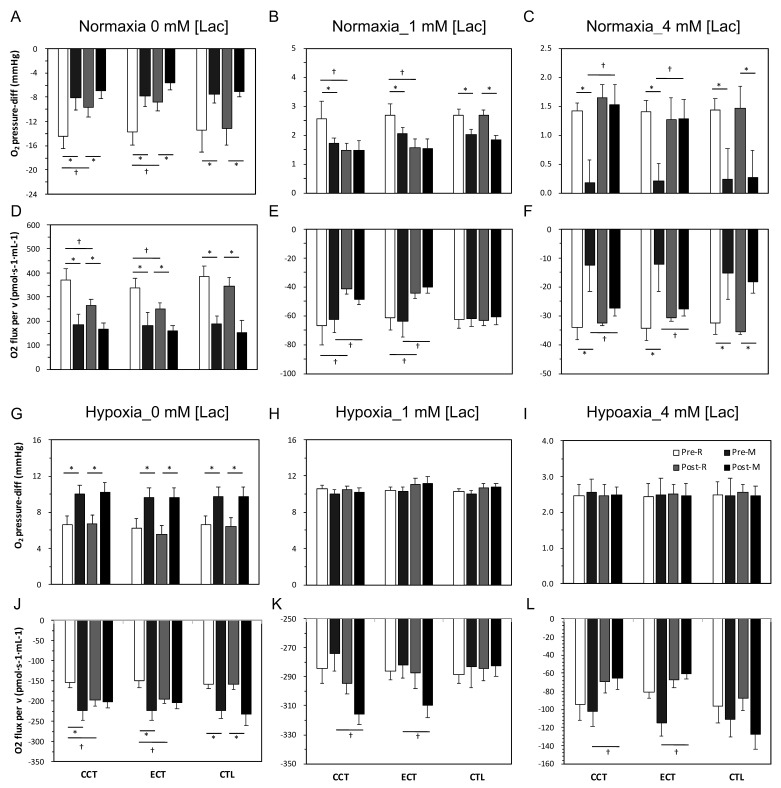
Measurement of oxygen release capacity of erythrocytes among three groups in normoxia and hypoxia conditions. Levels of O_2_ pressure-diff in normoxia condition: (**A**) at 0 mM [lac], (**B**) at 1 mM [lac], and (**C**) at 4 mM [lac]; oxygen flux per volume in normoxia condition: (**D**) at 0 mM [lac], (**E**) at 1 mM [lac], and (**F**) at 4 mM [lac]; levels of O_2_ pressure-diff in hypoxia condition: (**G**) at 0 mM [lac], (**H**) at 1 mM [lac], and (**I**) at 4 mM [lac]; oxygen flux per volume in hypoxia condition: (**J**) at 0 mM [lac], (**K**) at 1 mM [lac], and (**L**) at 4 mM [lac]; Pre, pre-intervention; Post, post-intervention; R, at rest; M, immediately after a GXT; * *p* < 0.05, R vs. M; † *p* < 0.05, Pre vs. Post. Values were mean ± SEM.

As Figure 5A,F shows, oxygen was absorbed into the cell when isolated erythrocytes were added to the normoxia chamber, which produced a negative oxygen pressure difference (O_2_ pressure-diff). Furthermore, this oxygen was released at lactate acid concentrations of 1 and 4 mM. After an acute GXT, the magnitudes of oxygen absorption and release were diminished (Figure 5A,C) and coupled with a reduced flux velocity (Figure 5D,F). Following the interventions, both training groups exhibited a diminished O_2_ pressure-diff and velocity in 0 and 1 mM lactate acid conditions at rest and even after the GXT. However, both CCT and ECT induced maintenance of these parameters at resting levels in the 4 mM lactate acid condition (Figure 5C,F).

### 3.6. Erythrocyte O_2_ Release Capacity in Hypoxia Conditions

O_2_ was released immediately (O_2_ pressure-diff was positive) from erythrocytes when erythrocytes were added to the hypoxia chamber, and the magnitude of release was further augmented due to the GXT (Figure 5G,L). An enhanced release velocity was also noticed (Figure 5J). After training, although no alternation in the oxygen release amount was observed at rest or even after the GXT, a faster velocity was observed in both the CCT and ECT groups under 0, 1 and 4 mM lactate acid (Figure 5J,K).

### 3.7. Relationships between GSSG/GSH and Lactate/Pyruvate and between Intracellular pH and Lactate Concentration

**Figure 6:**
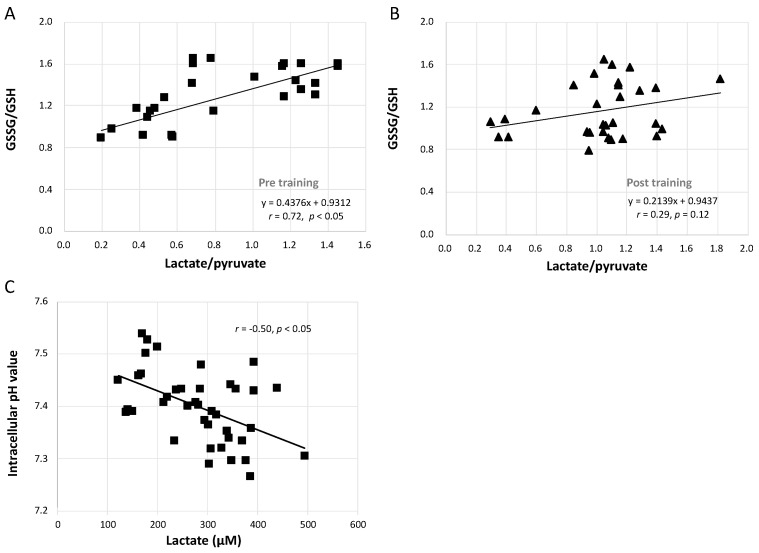
Correlation analysis between GSSG/GSH, lactate/pyruvate, intracellular pH value and lactate concentration. (**A**) relationship between GSSG/GSH and lactate/pyruvate before interventions, (**B**) relationship between GSSG/GSH and lactate/pyruvate after interventions, and (**C**) the correlation between intracellular pH value and lactate concentration. GSH, glutathione; GSSG, glutathione disulfide.

Figure 6A shows that the increase in the GSSG/GSH ratio caused by the GXT was significantly linearly related to the augmented lactate/pyruvate ratio (*r* = 0.72, *p* < 0.05). However, this correlation was blunted after training in both the CCT and ECT groups (Figure 6B, *r* = 0.29, *p* = 0.12). Furthermore, Figure 6C demonstrates that the lowered intracellular pH was moderately related to the greater lactate concentration (*r* = −0.50, *p* < 0.05).

## 4. Discussion

Erythrocyte metabolism includes glycolytic pathways producing both energy and oxidation–reduction intermediates that support O2 transport and antioxidative capacity. This study is the first to demonstrate that both CCT and power-matched ECT not only ameliorate antioxidation capacity in erythrocytes but also significantly increase the flux of anaerobic glycolysis to facilitate oxygen release efficacy. We further elucidated that the reduced oxygen affinity is due to greater lactate synthesis and not to the production of 2,3-BPG. Although ECT did not result in significant improvement in VO2 max, the im-proved VT performance indicates the positive effect ECT has on the aerobic capacity of young and sedentary men.

Several studies have reported that blood GSSG and thus the GSH/GSSG ratio decrease in response to acute exercise, and regular exercise may increase antioxidative capacity [28]. After both CCT and ECT, neither the GSSG/GSH ratio nor GSH decreased due to the GXT, while enhanced anaerobic glycolysis provided more precursors to activate the PPP. In addition, the downstream X5P and E4P, from the PPP back to glycolysis, also increased after training, thus suggesting significant enhancement of GSH biosynthesis under stress. The linear relationship between GSSG/GSH and lactate/pyruvate was disrupted after training, which may be due to a changed dominance of energy or/and antioxidant production [29]. In addition, the oxidative environment leads to the production of Fe3^+^ (met-Hb). To restore Hb function, met-Hb must be reduced mainly by NADH-dependent cytochrome b5 reductase [30].

An interesting aspect of the metabolic pathways is that intracellular pH (pHi) regulates both the glycolytic pathway and the PPP. As with glycolysis, the optimum activity of the enzymes driving the PPP occurs at an alkaline pHi [31]. Generally, the presence of NADPH blocks PPP negative feedback control and shifts metabolism from the PPP to glycolysis, thus increasing the formation of NAD^+^ [32]. Although NADH does not directly participate in the reduction of Fe3^+^ to Fe2^+^ in hemoglobin, it has the ultimate responsibility of providing the reducing power needed for such a reaction [33]. Under normal homeostasis in general, and especially in the case of the high glycolytic flux that is required during high-intensity exercise, lactate dehydrogenase oxidizes NADH back to NAD^+^ in the conversion of pyruvate to lactate, thereby maintaining necessary levels of the cofactor for the continuation of glycolysis. Cyclists in the high-class group had a higher posttest lactate/pyruvate ratio, which is proportional to NADH/NAD^+^ and a marker of glycolytic capacity [34]. Additionally, it has been confirmed that high glucose levels induce in-creases in lactate and 6PG production in vitro and ensure a longer supply of energy sources, preventing the loss of GSH [35].

The blood lactate that was progressively elevated with exercise intensity further reduces local blood pH and thus enhances the Bohr effect to attenuate O_2_ affinity and facilitate O_2_ release [17]. In contrast, pulmonary O_2_ uptake is enhanced, but muscle un-loading is hindered with high-affinity hemoglobin [36]. The capillary transit times were very limited; thus, the exchange speed is critical for evaluating the physiological fitness of erythrocytes [37]. Therefore, we developed a novel method for quantifying gas exchange in a constant number of erythrocytes and used it to assess the quality and quantity of O_2_ releasing capacity.

To clarify the oxygen release efficacy of erythrocytes, we measured the PO_2_, oxygen release/absorption velocity and acceleration under 0, 1, and 4 mM lactic acid concentrations to mimic resting and near AT conditions, respectively. The PO_2_ increased (oxygen release) when lactic acid was added. Even with a smaller amount of O_2_ being supplied to the tissue, the efficacy was enhanced with faster acceleration and velocity, which indicates better efficiency for release. The improved release of oxygen efficacy at 4 mM [lac] might be associated with improved cycling performance before reaching the anaerobic threshold. In addition, a diminished magnitude and velocity of O_2_ absorption after the acute GXT were noticed. We first speculated that this was a consequence of sufficiently oxygenated Hb after high oxygen demand activity or that this impaired quantity and quality may also be related to increases in oxidative and met-Hb levels after exhaustive exercise [38]. We further demonstrated an enhanced, strong correlation between lactate concentration and oxygen release magnitude and velocity under hypoxic conditions. The O_2_ affinity of athletes is lower than that of untrained subjects, which is consistent with our results [39]. Slow VO_2_ kinetics incur a high O_2_ deficit, usually resulting in poor exercise tolerance [40].

Lactic acid plays a vital indirect role in tissue O_2_ delivery apart from the direct allosteric interaction of lactate ions with Hb [41]. Lactic acid increases the Bohr shift via acidification as well as via liberation of CO_2_ [42]. Therefore, the lower affinity of hemoglobin for the O_2_ of erythrocytes in athletes at rest is maintained by the factor(s) dominating pH and lactate-driven regulation. Under heavy exercise (above the lactic acidosis threshold), acidification of muscle capillary blood by lactic acid accounts for virtually all of the oxygen unloaded from Hb [43].

Erythrocytes must be considered a potential storage site of lactate, storage of which leads to a greater gradient from the interstitial fluid to plasma. This mechanism improves the rate of release from muscle and ameliorates exercise performance [44]. However, a previous study demonstrated that the lactate distribution in erythrocytes and plasma after high-intensity running was not different between trained and untrained subjects. Hence, lactate uptake by erythrocytes cannot or can only in part be seen as a contributor to aerobic athletic performance [45]. Traditionally, a higher Bohr effect is supposed to be related to a higher 2,3-BPG in erythrocytes [46]. However, the generation of 2,3-BPG results in the overall production of ATP per mole of glucose is decreased to zero. Therefore, accumulation of 2,3-BPG leads to decreased production of 2,3-BPG by competitive feedback inhibition of di-phosphoglycerate mutase [47]. The relative ratio of 2,3-BPG synthase to 2,3-BPG phosphatase decreased dramatically with decreasing pH value [48]. The lactate effect even increased after 2,3-BPG depletion [49]. In this study, the presence of large lactate concentrations leading to lower pH values may effectively limit the production of 2,3-BPG [50]. When the downstream enzymes of 2,3-BPG, such as pyruvate kinase and lactate dehydrogenase, maintained higher activities, the enzyme activities of other pathways were significantly repressed [48]. Therefore, this reversed flux of the 2,3-BPG shunt is crucial in maintaining the activities of the latter part of glycolysis and the production of ATP in the latter half of the storage period.

The results in this study clearly presented that, although both ECT and CCT ameliorate the erythrocyte antioxidant and oxygen releasing capacity, thus further delays the anaerobic threshold, yet only ECT has significantly less cardiopulmonary stress without undesirable fatigue or pain impact during the whole training period. Therefore, we suggest that ECT is preferred to those who have exercise intolerance or low physical activity, whereas CCT may be more feasible for those who have general physical activity to increase the ability to cope with the physical demands of daily activity. These findings provide a new suggestion on why ECT is worthy to further developed as a suitable training strategy in cardiopulmonary rehabilitation or the elderly.

A small sample size (*n* = 12 in each group) is a major limitation of this study. However, the results for aerobic capacity and the novel interpretation of the O_2_ release and antioxidative mechanisms in the metabolic pathways obtained from this investigation have high statistical power (0.862 to 1.000). We speculate that metabolic alteration of erythrocytes generates higher ATP concentrations followed by lactate production. We did not directly detect the ATP concentration because of the fast rate of ATP hydrolysis. Therefore, in this study, we indirectly inferred ATP demand by the lactate/pyruvate ratio. We did not see potential model alterations in Hb affinity as blood traverses the exercising muscles in accordance with local changes in temperature, pHi, or CO_2_. There are certainly differences between and challenges with in vivo vs. in vitro measurements of O_2_ dissociation curve dynamics in both the lungs and muscles in response to variables such as temperature, pHi, and 2,3-BPG. A few studies have shown that the additive effects of temperature and pH are responsible for shifting the O_2_ dissociation curve affinity, especially with prolonged exercise [51]. Additionally, the subjects tended to be young and healthy; thus, further clinical evidence is still required to extrapolate the present results to patients with hemorheological or hemodynamic disorders.

Although the glutathione system is a principal nonenzymic antioxidant system in erythrocytes yet, GSSG is rapidly formed, but it quickly disappears once the oxidative stimulus is interrupted; conversely, S-glutathionylated proteins (PSSGs) may be produced more slowly but are more durable [52]. Therefore, the PSSGs is a worthy parameter for further investigation [53]. In addition, although previously study suggested that exercise-induced changes in the nonenzymatic glutathione system seem to be more effective in erythrocytes [54]. Nevertheless, many studies have indicated the activity of glutathione peroxidase (GPx) plays a key component in the antioxidant experiment [55], and regular cardiovascular training increased GPx activity in skeletal muscle [56]. Taking together, both PSSGs and the immunoblotting for GPx are very used to supply important information on the state of this antioxidant network in the future. To assess the reliabilities of biomarkers, metabolites and oxygen releasing capacity to exercise, the subjects (*n* = 5) in a prior study were tested twice at two-day intervals. Results of responses to exercise were highly reproducible from day to day. The intraclass correlation coefficients were from 0.811 to 0.954. Additionally, it requires separate analytical measurements for GSH and GSSG for accurate analysis and specific methodological procedures needed to detect samples [57]. Although the use of classical and well-validated in previous studies [26,58], techniques to perform our measurements, requiring immediate and complex processing of blood samples [59]. This limits the possibility of receiving samples from different centers to be analyzed. In the present study, we tested our participants at the same time of the day and asked them to record their nutritional intake and to maintain the same diet (data not shown). Thus, we assume that our results well represent the physical adaptations after exercise training. Importantly, other more adequate and precise methodologies should be considered in future studies [53].

## 5. Conclusions

**Figure 7:**
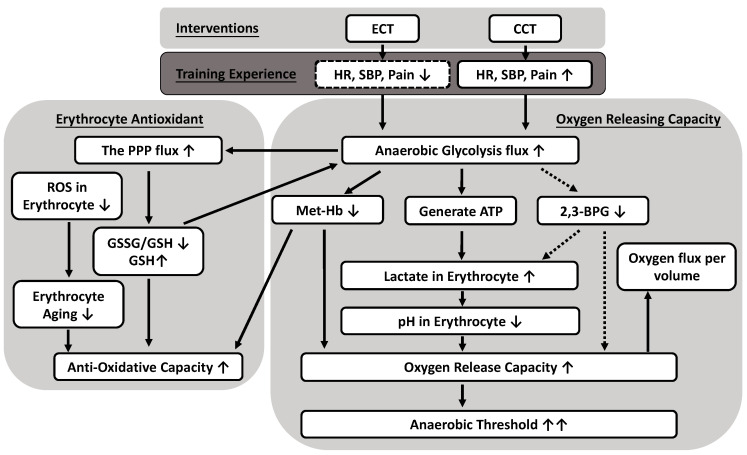
Possible mechanisms of anaerobic glycolytic pathways producing both oxygen releasing and antioxidative capacity caused by eccentric (ECT) and concentric cycling training (CCT). Both CCT and ECT at a given workload enhance an anaerobic glycolysis flux to ameliorate antioxidative capacity in erythrocytes, as well as significantly facilitate oxygen release efficacy. The reduced oxygen affinity is due to greater lactate synthesis and lower intracellular pH, instead of the production of 2,3-BPG. Solid line: positive regulation; dotted line: negative regulation.

This study presented evidence that both ECT and CCT simultaneously promote flux into the pentose phosphate pathway and anaerobic glycolysis pathway in response to overcoming accumulated oxidative stress and regulating internal O_2_ dissociation, respectively. The adaptations of the metabolite process not only increased the synthesis of GSH but also enhanced the production of lactate in glucose metabolism in trained erythrocytes. The lower intracellular pH value related to lactate, instead of 2,3-BPG, ameliorated the O_2_ release efficacy of erythrocytes under different O_2_ gradients. In addition, the reduced amount of met-Hb also contributed to the O_2_ release. The above experimental findings reflect many positive effects of both interventions and provide a novel interpretation of delayed anaerobic threshold by ameliorated O_2_ release and antioxidative mechanism in the metabolic pathway (Figure 7). Therefore, ECT is a feasible and promising exercise regimen that promotes a less cardiovascular stress way to exercise without undesirable fatigue impact and provides important implications for those who have exercise intolerance.

## Figures and Tables

**Table 1:** Anthropometric data and ventilatory responses to graded exercise test in concentric and eccentric training groups.

		CCT		ECT		CTL	
		Pre	Post	Pre	Post	Pre	Post
**Anthropometrics Characteristics**							
Age, year		21.3 ± 0.5	—	21.7 ± 0.4	—	21.6 ± 0.6	—
Height, cm		174 ± 1	—	173 ± 2	—	175 ± 1	—
Weight, kg		67.5 ± 2.3	68.4 ± 1.9	68.1 ± 1.3	67.4 ± 1.5	67.2 ± 2.2	68.0 ± 2.2
**Hematological Parameters**							
Red blood cells, 10^6^/µL		5.10 ± 0.08	5.05 ± 0.05	5.13 ± 0.06	5.06 ± 0.07	5.13 ± 0.06	5.13 ± 0.07
Hb, g/dL		14.9 ± 0.2	14.5 ± 0.2	15.0 ± 0.4	14.9 ± 0.3	15.0 ± 0.3	14.7 ± 0.3
Hematocrit, %		45.4 ± 0.7	44.5 ± 0.6	46.2 ± 0.6	45.3 ± 0.6	46.2 ± 0.6	45.2 ± 0.5
i-**STAT Parameters**							
Blood pH, unit	Rest	7.37 ± 0.02	7.37 ± 0.01	7.36 ± 0.01	7.37 ± 0.01	7.36 ± 0.02	7.35 ± 0.01
	Ex	7.23 ± 0.02 #	7.21 ± 0.02 #	7.19 ± 0.01 #	7.19 ± 0.01 #	7.19 ± 0.03 #	7.19 ± 0.02 #
Blood lactate, mM	Rest	0.88 ± 0.11	0.87 ± 0.11	0.87 ± 0.06	0.98 ± 0.08	0.89 ± 0.09	0.93 ± 0.11
	Ex	13.00 ± 0.59 #	12.66 ± 0.64 #	13.99 ± 0.51 #	13.9 ± 0.49 #	13.16 ± 0.69 #	12.38 ± 0.73 #
**Ventilation Threshold**							
Work-rate, watt		125 ± 6	151 ± 6 * †	120 ± 4	136 ± 5 *	121 ± 4.3	122 ± 6.8
V_E_, L/min		44.8 ± 2.3	52.3 ± 2.7 *	43.5 ± 1.9	51.8 ± 4.3 *	45.2 ± 1.8	46.2 ± 3.9
VO_2_, mL/min/kg		21.3 ± 0.8	26.4 ± 1.0 * †	21.3 ± 0.6	23.3 ± 0.5 *	21.6 ± 1.1	21.2 ± 1.2
% of VO_2max_, %		59.8 ± 2.0	66.1 ± 2.0 *	60.6 ± 1.7	67.5 ± 1.2 *	60.92 ± 2.1	61.16 ± 1.9
**Maximal Exercise Performance**							
Work-rate, watt		191 ± 3	223 ± 5 * †	189 ± 4	201 ± 5 *	188 ± 5	190 ± 5
V_E_, L/min		107.4 ± 3.2	118.8 ± 2.5 *	111.9 ± 3.7	115.3 ± 2.2	109.95 ± 4.7	108.3 ± 4.3
VO_2_, mL/min/kg		35.7 ± 1.1	40.0 ± 0.8 *	35.2 ± 0.7	34.6 ± 0.7	34.1 ± 1.0	34.6 ± 1.5
OUES, unit		814 ± 23	886 ± 20 *	817 ± 16	829 ± 24	816 ± 19	825 ± 20
V_E_-VCO_2_ slope, unit		36.8 ± 1.5	36.8 ± 1.6	37.6 ± 1.9	38.5 ± 2.4	35.7 ± 1.6	35.7 ± 1

Values were mean ± SEM. **Hb**, hemoglobin; **V_E_**, minute ventilation; **VO_2_**, oxygen consumption; **OUES**, oxygen uptake efficiency slope; **CCT**, concentric cycling training; **ECT**, eccentric cycling training; **CTL**, control group. **Pre**, pre-intervention; **Post**, post-intervention; **Rest,** at rest; **Ex,** immediately after the GXT; # *p* < 0.05, Rest vs. Ex; * *p* < 0.05, Pre vs. Post; † *p* < 0.05, CCT vs. ECT.

## Data Availability

All data is contained within the article.

## Supplementary Materials

The following are available online at https://www.mdpi.com/2076-3921/10/2/285/s1, Figure S1: the scheme of testing oxygen release capacity in erythrocytes by using high-resolution respirometry.
