# Supplementary material for: Eccentric Cycling Training Improves Erythrocyte Antioxidant and Oxygen Releasing Capacity Associated with Enhanced Anaerobic Glycolysis and Intracellular Acidosis
Source: Antioxidants (Basel). 2021 Feb 13;10(2):285. doi: 10.3390/antiox10020285 (PMC7918820; doi:10.3390/antiox10020285)

Figure S1

The scheme of testing oxygen release capacity in erythrocytes by using high-resolution respirometry. **(A)** Hypoxia chamber, and **(B)** normaxia chamber. The red line indicated the oxygen oxygen release/uptake velocity, the blue line indicated the oxygen pressure.

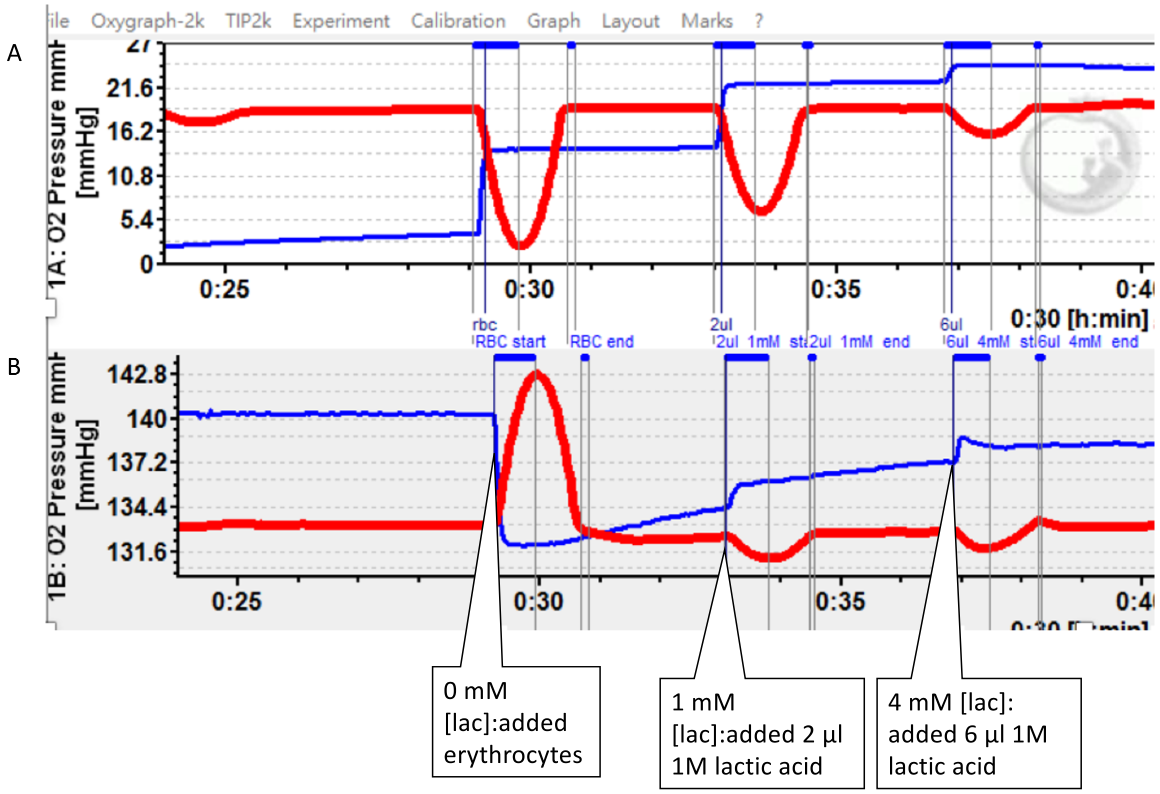

Supplement: Supplementary file 1 [file antioxidants-10-00285-s001.pdf]
